# Caffeic Acid Derivatives from *Eupatorium perfoliatum* L.

**DOI:** 10.3390/molecules14010036

**Published:** 2008-12-23

**Authors:** Mareike Maas, Frank Petereit, Andreas Hensel

**Affiliations:** University of Münster, Institute for Pharmaceutical Biology and Phytochemistry (IPBP), Hittorfstraße 56, D-48149 Münster, Germany

**Keywords:** *Eupatorium perfoliatum* L., Asteraceae, Flavonoids, Dicaffeoylglucaric acid, NMR.

## Abstract

From the ethyl acetate soluble fraction of a methanol/water extract of the herb *Eupatorium perfoliatum* L. (Asteraceae) six caffeic acid derivatives have been isolated and identified by 1D- and 2D-NMR spectroscopic data. Besides the common quinic acid derivatives 5-caffeoylquinic acid (chlorogenic acid), 3-caffeoylquinic acid (neochlorogenic acid) and 3,5-dicaffeoylquinic acid, three up to now unknown depsides of caffeic acid with glucaric acid have been isolated: 2,5-dicaffeoylglucaric acid, 3,4-dicaffeoylglucaric acid, and 2,4- or 3,5-dicaffeoylglucaric acid.

## Introduction

*Eupatorium perfoliatum* L., Asteraceae, is a medicinal herb from the Northern American continent known as boneset or thoroughwort. It has been extensively used by the native inhabitants, especially for the treatment of fever and flu. European settlers adopted the use of the herb which led to a monograph in the USP and NF in the 19^th^ and beginning of 20^th^ century [1,2,3,4]. Today *E. perfoliatum* is mainly used in central Europe as an immunostimulating remedy. 

*E. perfoliatum* is phytochemically characterized by the occurrence of flavonoids [5,6,7], sesquiterpene lactones [8,9,10], triterpenes and steroids [11, 12], and by small amounts of volatile oil [13]. An alkaline-soluble polysaccharide fraction was shown to exhibit immunostimulating activities under *in vitro* conditions [14,15,16]. Furthermore, investigations concerning antiinflammatory [17, 18], cytotoxic and antibacterial [19] as well as antiprotozoal [20] activities have been carried out. The few clinical studies dealing with different preparations of *E. perfoliatum* only partly support the phytotherapeutic indications [21,22,23]. For a review on *E. perfoliatum* see also Maas and Hensel [24].

This study deals with the characterisation and isolation of phenolic compounds in the herb of *E. perfoliatum* with a focus on hydroxycinnamic acid derivatives. These substances have not been investigated in this plant so far. The study led to the description of three up to now unknown caffeoylglucaric acid derivatives, besides some commonly occurring caffeoylquinic acids, e. g. chlorogenic acid.

## Results and Discussion

**Figure 1:**
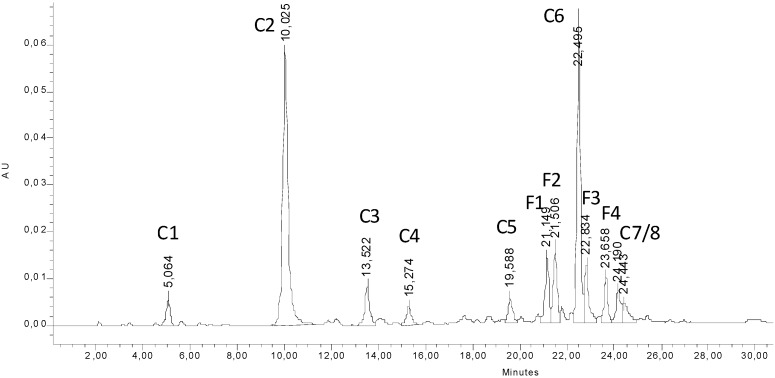
RP-HPLC chromatogram of the ethyl acetate fraction obtained from a partition of a MeOH/water extract from *E. perfoliatum*; detection wavelength 325 nm. Peaks **C1** to **C8** correspond to caffeic acid derivatives, peaks **F1** to **F4** correspond to flavonoids.

A crude methanol/water (70/30 V/V) extract from the dried aerial parts of *E. perfoliatum* was partitioned between ethyl acetate and acidic aqueous solution to enrich the hydroxycinnamic acid derivatives as well as the flavonoid glycosides in the ethyl acetate fraction. The ethyl acetate fraction was analysed by HPLC, resulting in typical chromatograms as presented in Figure 1. 

By analysing the recorded DAD-UV spectra, eight of the twelve numbered peaks were assigned to hydroxycinnamic derivatives (**C1** to **C8**, λ_max_ at approx. 325 nm) and four to flavonoids (**F1** to **F4**, λ_max_ at approx. 354 nm). The four flavonoid glycosides were identified by comparison of retention times and spiking experiments with the respective reference standards as hyperoside (quercetin-3-O-β-galactoside, **F1**), isoquercitrin (quercetin-3-O-β-glucoside, **F2**), trifolin (kaempferol-3-O-β-galactoside, **F3**) and astragalin (kaempferol-3-O-β-glucoside, **F4**). The assignment was confirmed by TLC.

Isoquercitrin and trifolin have not been reported as constituents of *E. perfoliatum* up to now, whereas astragalin and hyperoside, as well as the aglyca kaempferol and quercetin have been described by Wagner *et al*. [6] and Habtemariam [5].

To clarify the structural features of the caffeic acid compounds the ethyl acetate extract was subsequently fractionated on a preparative scale on Sephadex^®^ LH-20, followed by different purification steps using fast centrifugal partition chromatography (FCPC), medium pressure liquid chromatography (MPLC) and preparative HPLC (see Experimental section). The five hydroxycinnamic acid derivatives **C1** to **C5** were isolated as pure compounds and identified by the respective ^1^H- and ^13^C-NMR spectra, combined with ^1^H-^1^H-correlated spectroscopy (COSY), heteronuclear single quantum coherence spectroscopy (HSQC) and heteronuclear multiple bond correlation spectroscopy (HMBC). 

The two main constituents of the ethyl acetate fraction, **C2** and **C6**, were identified as 5-O-caffeoyl-quinic acid (chlorogenic acid) and 3,5-O-dicaffeoylquinic acid by ^1^H-NMR spectroscopy (data not shown) and comparison with recorded data [25, 26]. Peak **C1** proved to be a structural analogue of chlorogenic acid, which by comparison with known spectra was identified as 3-O-caffeoylquinic acid (neochlorogenic acid) [25]. 

The minor peaks **C7** and **C8** eluted in the same Sephadex^®^ LH-20 fraction as **C6**. The UV spectra were typical for hydroxycinnamic acid derivatives, and because of the similar chromatographic behaviour of these three substances, **C7** and **C8** were also tentatively identified as dicaffeoylquinic acid derivatives. Because of the limited amounts a detailed NMR study was not possible.

The ^1^H-NMR spectra of **C3**, **C4** and **C5** all showed the typical signal sets (aromatic AMX spin system, olefinic side chain) of two caffeic acids in the region between 6 and 8 ppm, indicating the presence of two inequivalent caffeoyl moieties in each compound. In the region between 4 and 6 ppm four signals appeared, two *d* and two *dd*, of which in each case two were shifted downfield, indicating the site of esterification with caffeic acid. In **C3** one *d* and one *dd*, in **C4** the two *dd*, and in **C5** and the two *d* were shifted downfield. Further NMR studies including ^13^C, H-H-COSY, HSQC and HMBC indicated the presence of a hexaric acid as alcoholic moiety, esterified with two caffeoyl rests each.

Hexaric acid itself has six diastereomers of which four are symmetric (allaric, mannaric, idaric and galactaric acid) and two asymmetric (altraric and glucaric acid). **C3** showed a asymmetric esterification pattern (positions 2,4 or 3,5), so the caffeic acid signals could be duplicated with any of the possible hexaric acids. To determine the existing diastereomer, compound **C3** was hydrolysed under alkaline conditions [27]. Shortly afterwards a ^1^H-NMR spectrum of the reaction mixture was recorded and the signals related to the hexaric acid were compared with published spectra [27]. The hydrolysed hexaric acid showed four inequivalent signals, indicating the existence of an asymmetric acid, which was further identified as glucaric acid. From the NMR data it cannot be distinguished whether **C3** is the 2,4- or the 3,5-dicaffeoylglucaric acid. As the rotational conformation of the molecule is not fixed, it is not possible to obtain information about the dihedral angles by the coupling constants. For the same reason circular dichroism experiments will not be successful. Perhaps x-ray structure analysis will help to determine the absolute stereochemistry. Up to this point **C3** can only be referred to as 2,4- or 3,5-dicaffeoylglucaric acid.

**Figure 2:**
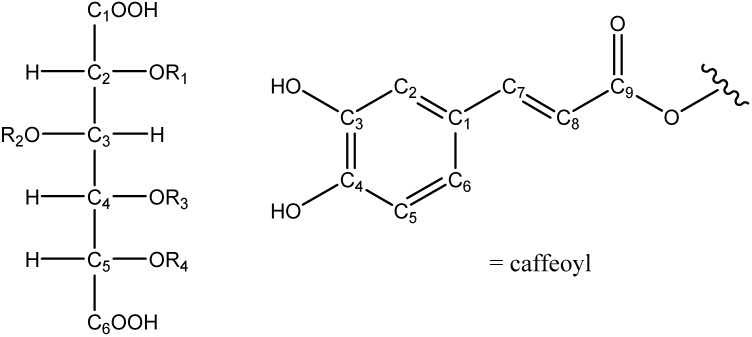
Structure of dicaffeoylglucaric acid derivatives **C3**, **C4** and **C5** from *E. perfoliatum*.

For compounds **C4** and **C5**, with a symmetric substitution pattern (positions 3,4 and 2,5, resp.), a symmetric hexaric acid had led to two equivalent caffeic acid moieties with only one set of signals. As the caffeic acid signals were doubled in both cases, only altraric or glucaric acid are possible as the hexaric acid component. For analogy reasons, these two compounds were identified as 3,4-dicaffeoylglucaric acid (**C4**) and 2,5-dicaffeoylglucaric acid (**C5**). The structures of compounds **C3**, **C4** and **C5** are shown in Figure 2.

**C3:** R_1_ = R_3_ = caffeoyl, R_2_ = R_4_ = H or R_2_ = R_4_ = caffeoyl, R_1_ = R_3_ = H

**C4:** R_2_ = R_3_ = caffeoyl, R_1_ = R_4_ = H

**C5:** R_1_ = R_4_ = caffeoyl, R_2_ = R_3_ = H

Depsides of caffeic acid and quinic acid are widespread throughout all kind of plants. Remarkably high concentrations of chlorogenic acid and caffeic acid can be found in the green seeds of *Coffea arabica* and *canephora*. Many members of the family Asteraceae are also known to contain caffeoylquinic acid derivatives, e. g. mountain arnica (*Arnica montana*) or artichoke (*Cynara scolymus*). This group of compounds exhibit antioxidative and antiinflammatory properties; the dicaffeoylquinic acids in artichoke leaves are part of the active ingredients responsible for the choleretic effect [28]. Caffeoylquinic acids are known compounds in other *Eupatorium* species [29,30,31], but have not been described for *Eupatorium perfoliatum* before. 

In contrast to these common compounds depsides of hydroxycinnamic acids with hexaric acids are very rarely described. The first description of monocaffeoylglucaric acid was published for tomato leaves (*Lycopersicon esculentum*) [32], followed by a report for the same derivative from *Cestrum euanthes*, both from the Solanaceae family [33]. Depsides of coumaric and ferulic acid with glucaric and galactaric acid were found both in *Secale cereale*, Poaceae [34] and *Citrus* species, Rutaceae [35, 36]. Two reports describe the occurrence of hydroxycinnamoylhexaric acid derivatives in the Asteraceae family: in yacon root (*Smallanthus sonchifolius*) 2,4- or 3,5-dicaffeoyl-, 2,5-dicaffeoyl- and 2,3,5- or 2,4,5-tricaffeoylaltraric acid have been found [27], and 2-[(3S)-3-hydroxybutanoat]-3,4,5-tricaffeoylglucaric acid and 3,4,5-tricaffeoylglucaric acid (Leontopodic acid and Leontopodic acid B) have been isolated in edelweiss (*Leontopodium alpinum*) [37, 38]. Concerning the two Leontopodic acids it is not clear why the authors excluded the 2,3,4-tricaffeoylglucaric acid derivatives as possible conformational isomers.

Strack *et al*. [39] discovered the biochemical relationship of chlorogenic acid and caffeoylglucaric acid in tomato cotyledons: caffeic acid is transmitted from chlorogenic acid to glucaric acid by a specific enzyme. So it can be assumed that the occurrence of chlorogenic acid is a precondition for the development of caffeoylhexaric acid derivatives, but a special enzymatic pathway is needed to perform the transformation. These enzymes obviously seem to be very uncommon.

The hexaric acid derivatives of edelweiss and yacon have been shown to exhibit antioxidative and radical scavenging activities [37, 40] and the caffeoylglucaric acid from tomato possesses inhibiting activity against the tomato fruit worm [32]. Beside these data, nothing is known about possible pharmacological properties of this group of substances.

The three dicaffeoylglucaric acid derivatives identified in the present study (3,4-dicaffeoylglucaric acid, 2,5-dicaffeoylglucaric acid and 2,4- or 3,5 dicaffeoylglucaric acid) have not been reported before. Therefore they are promising substances for a further research on the activity determining constituents of *E. perfoliatum.*

Because of their rare occurrence, these glucaric acid derivatives can be useful for identity control of herbal material of *E. perfoliatum*. In the respective official monograph of the homeopathic pharmacopoeia [41] no unambiguous chromatographic data for quality control, especially for identification, are published. After validation, the analytical HPLC method described in this report and based on the identification of four flavonoids and several hydroxycinnamic acid derivatives may be a useful tool, not only for identity but also for stability control because these compounds tend to exhibit fast degradation by oxidative processes when stored under humid and oxidative conditions. 

## Experimental

### General

NMR spectra were recorded on a Varian AS 400 Mercuryplus spectrometer. Mass spectra were obtained on a Finnigan MAT mass spectrometer with electrospray ionisation. Optical rotations were measured with a Perkin-Elmer 341 digital polarimeter in methanol. Analytical HPLC was performed on a ProSep C18-5 mm column (Latek, Eppelheim, Germany), with a ternary gradient containing acetonitrile, methanol and 0.1% trifluoroacetic acid in aqueous solution [26]. The Waters 996 photodiode array detector was set at 325 nm (λ_max_ of caffeic acid derivatives). TLC Silica gel 60 F_254_ on Aluminium sheets (Merck, Darmstadt, Germany) was used for analytical TLC with ethyl acetate/water/formic acid (90:5:5) as mobile phase. Hydroxycinnamic acid derivatives were detected by a blue fluorescence at 366 nm and a greenish-blue fluorescence at 366 nm after spraying with diphenylboryloxyethylamine in methanol (”Naturstoffreagenz A“). Flavonoid glycosides were detected as light orange spots at 366 nm after spraying with the reagent. Reference substances were taken from the reference library of the Institute for Pharmaceutical Biology and Phytochemistry, Münster. The hydrolysis of 2,4-/3,5-dicaffeoylglucaric acid was performed according to Takenaka *et al*. [27].

### Plant material

Dried *Eupatorium perfoliatum* herb (Lot 3458) was purchased from Alfred Galke, Gittelde, Germany. A voucher specimen has been deposited in the documentation file of the Institute of Pharmaceutical Biology and Phytochemistry (EP-1). The material was grown in summer 2005 in northern parts of the United States of America (U.S.A.) under continental climate conditions. The material was delivered in January 2006 to Germany and was obtained as bulk material from the supplier. The material was investigated according the specifications of the official Homeopathic Pharmacopoeia HAB 2000, Germany. All results obtained fulfilled the respective specifications of the official Pharmacopoeia, therefore batch 3458 is deemed to be a representative lot. 

### Extraction and isolation

The dried and cut plant material (1 kg) was extracted with cold methanol/water (70/30 V/V, approx. 20 L) by rotor-stator extraction (Ultra-Turrax^®^). The combined extracts were concentrated to about 1 L of aqueous solution, and filtered to remove the precipitated chlorophyll. Partition between aqueous solution and dichloromethane was used to further remove lipophilic substances. The aqueous solution was acidified to pH 2 with 1 N HCl and partitioned with ethyl acetate. After removal of the solvents, the residues were lyophilized to yield 76 g H_2_O-soluble fraction (W) and 16 g EtOAc-soluble fraction (E). E was further fractionated by column chromatography with Sephadex^®^ LH-20 (730 x 55 mm inner diameter) using methanol/water 50/50 (13 L), followed by acetone/water 7/3 (2.5 L) to yield 20 fractions. Fractions were monitored by TLC and characterised by analytical HPLC.

Fraction 9 (1,270 mg) was analysed directly, leading to the identification of chlorogenic acid (5-caffeoylquinic acid, **C2**). Fractions 7 (320 mg), 12 (160 mg) and 20 (1,300 mg) were fractionated by fast centrifugal partition chromatography (FCPC), performed on a CPC Kromaton (Angers, France) with a 200 mL rotor. For fractions 7 and 12 a solvent system consisting of ethyl acetate/acetone/diluted HCl (pH 2) 1/1/1 (V/V/V) was used, for separation of fraction 20 the solvent system was n-heptane/ethyl acetate/methanol/acetic acid 1 % 1/2/1/2 (V/V/V/V). In all cases the upper phase served as mobile phase (ascending mode). 3-caffeoylquinic acid (**C1**, 50 mg) was isolated from fraction 7, while fraction 20 yielded 3,5-dicaffeoylquinic acid (**C6**, 208 mg). 2,4- or 3,5-dicaffeoylglucaric acid (**C3**, 46 mg) was purified out of a subfraction of fraction 12 by preparative HPLC (Hypersil ODS 5 µm, 250 mm x 16 mm inner diameter, gradient with acetonitrile and 1 % trifluoroacetic acid in aqueous solution, UV detector set at 325 nm, flow 5 ml/min). 

Fractions 15 (640 mg) and 16 (820 mg) were separated by MPLC (Spot Liquid Chromatography Flash from Armen Instrument, France) with a methanol/water gradient on a RP-18 phase (SVF D26 – RP 18, 40-63 µm, 37 g, Merck Chimie SAS). 3,4-Dicaffeoylglucaric acid (**C4**, 10 mg) was obtained from fraction 15, while fraction 16 contained 2,5-dicaffeoylglucaric acid (**C5**, 33 mg). 

### 2,4-/3,5-Dicaffeoylglucaric acid *(**C3**)*

[α]^20^*_D_* = -54.56° (*c* = 0.0011 g∙mL^-1^ in MeOH); ESI-MS: [M - H]^-^ *m/z* 533; NMR (atom numbers with ´ or ´´ refer to the caffeoyl moieties):^ 1^H-NMR (methanol-D_3_, 400 MHz, CD_3_OH = 3.31 ppm,): δ 4.42 (*d*, J = 1.9 Hz, H-2 or H-5), δ 4.71 (*dd*, J = 2.4 and 3.4 Hz, H-3 or H-4), δ 5.34 (*d*, J = 1.6 Hz, H-2 or H-5, esterified with caffeic acid), δ 5.46 (*dd*, J = 4.4 and 6.4 Hz, H-3 or H-4, esterified with caffeic acid), δ 6.26/6.29 (*d*, J = 16.0 Hz, H-8´ and H-8´´), δ 6.67/6.74 (*d*, J = 8.2, H-5´ and H-5´´), δ 6.83/6.88 (*dd*, J = 2.0 and 8.2, H-6´ and H-6´´), δ 6.97/6.97 (*d*, J = 1.9 Hz, H-2´ and H-2´´), δ 7.50/7.60 (*d*, J = 15.9 Hz, H-7´ and H-7´´); ^13^C-NMR (CD_3_OD, 100 MHz, CD_3_OD = 49,15 ppm): δ 70.19 (C-3 or C-4), δ 71.21 (C-2 or C-5), δ 75.67 (C-2 or C-5, esterified with caffeic acid), δ 75.89 (C-3 or C-4, esterified with caffeic acid), δ 114.53/115.17 (C-8´ and C-8´´), δ 115.38/115.66 (C-2´ and C-2´´), δ 116.51 (C-5´ and C-5´´), δ 123.16/123.32 (C-6´ and C-6´´), δ 127.80/127.82 (C-1´ and C-1´´), δ 146.81/146.90 (C-3´ and C-3´´), δ 148.00/148.16 (C-7´ and C-7´´), δ 149.59 (C-4´ and C-4´´), δ 168.45/168.58 (C-9´ and C-9´´), δ 171.62 (C-1 or C-6), δ 175.11 (C-1 or C-6).

### 3,4-Dicaffeoylglucaric acid *(**C4**)*

[α]^20^*_D_* = -161.47° (*c* = 0.0010 g∙mL^-1^ in MeOH); ESI-MS: [M - H]^-^ *m/z* 533; NMR (atom numbers with ´ or ´´ refer to the caffeoyl moieties): ^1^H-NMR (methanol-D_3_, 400 MHz, CD_3_OH = 3.31 ppm): δ 4.53 (*d*, J = 2.2 Hz, H-2 or H-5), δ 4.64 (*d*, J = 1.6 Hz, H-2 or H-5), δ 5.79 (*dd*, J = 2.4 and 8.0 Hz, H-3 or H-4), δ 5.99 (*dd*, J = 2.0 and 8.0 Hz, H-3 or H-4), δ 6.16/6.18 (*d*, J = 15.8 Hz, H-8´ and H-8´´), δ 6.73/6.76 (*d*, J = 2.6 Hz, H-5´ and H-5´´), δ 6.85/6.88 (*dd*, J = 2.0 and 4.0 Hz, H-6´ and H-6´´), δ 6.97/7.00 (*d*, J = 1.6 Hz, H-2´ and H-2´´), δ 7.48/7.52 (*d*, J = 15.8 Hz, H-7´ and H-7´´); ^13^C-NMR (CD_3_OD, 100 MHz, CD_3_OD = 49.15 ppm): δ 71.39 (C-2 or C-5), δ 71.84 (C-2 or C-5), δ 74.33 (C-3 or C-4), δ 75.45 (C-3 or C-4), δ 114.76/114.98 (C-8´ and C-8´´), δ 115.19/115.32 (C-2´ and C-2´´), δ 116.42 (C-5´ and C-5´´) δ 123.25/123.35 (C-6´ and C-6´´), δ 128.14 (C-1´ and C-1´´), δ 146.97 (C-3´ and C-3´´), δ 147.86/148.01 (C-7´ and C-7´´), δ 149.66 (C-4´ and C-4´´), 168.23/168.67 (C-9´ and C-9´´), 174.69 (C-1 and C-6).

### 2,5-Dicaffeoylglucaric acid *(**C5**)*

[α]^20^*_D_* = -39.36° (*c* = 0.0010 g∙mL^-1^ in MeOH); ESI-MS: [M - H]^-^ *m/z* 533; NMR (atom numbers with ´ or ´´ refer to the caffeoyl moieties): ^1^H-NMR (methanol-D_3_, 400 MHz, CD_3_OH = 3.31 ppm): δ 4.22 (*dd*, J = 5.2 and 5.2 Hz, H-3 or H-4), δ 4.25 (*dd*, J = 4.6 and 4.6 Hz, H-3 or H-4), δ 5.14 (*d*, J = 5.7 Hz, H-2 or H-5), δ 5.32 (*d*, J = 4.2 Hz, H-2 or H-5), δ 6.33/6.35 (*d*, J = 15.4 Hz, H-8´ and H-8´´), δ 6.76/6.78 (*d*, J = 3.3 Hz, H-5´ and H-5´´), δ 6.94/6.97 (*dd*, J = 2.0 and 6.4, C-6´ and C-6´´), δ 7.06/7.07 (*d*, J = 2.0 Hz, C-2´ and C-2´´), δ 7.62/7.66 (*d*, J = 15.9 Hz, C-7´ and C-7´´); ^13^C-NMR (CD_3_OD, 100 MHz, CD_3_OD = 49,15 ppm): δ 72.17 (C-3 and C-4), δ 75.07 (C-2 and C-5), δ 114.61/114.92 (C-8´ and C-8´´), δ 115.32 (C2´ and C2´´), δ 116.63 (C-5´ and C-5´´), δ 123.30/123.39 (C-6´ and C-6´´), δ 127.90/128.02 (C-1´ and C-1´´), δ 146.98 (C-3´ and C-3´´), δ 147.97 (C-7´ and C-7´´), δ 149.89 (C-4´ and C-4´´), δ 168.27/168.63 (C-9´ and C-9´´), signals for C-1 and C-6 are missing.
